# Host Immune Responses and Pathogenesis to *Brucella* spp. Infection

**DOI:** 10.3390/pathogens10030288

**Published:** 2021-03-03

**Authors:** Sergio C. Oliveira

**Affiliations:** Department of Biochemistry and Immunology, Institute of Biological Sciences, Federal University of Minas Gerais, Belo Horizonte, Minas Gerais 31270-901, Brazil; scozeus1@gmail.com

Brucellosis, caused by the facultative intracellular bacteria *Brucella* species, is one the most prevalent zoonoses worldwide. *Brucella* causes >500,000 human infections per year, and brucellosis is underreported in endemic areas [1]. Between livestock losses and human morbidity, brucellosis imposes significant economic impacts, perpetuating poverty in endemic regions [2]. There is a considerable amount of evidence that indicates the capacity of *Brucella* sp. to avoid or interfere with components of the host immune responses, which plays a critical role in their virulence. It has been suggested that *Brucella* has developed a stealth strategy through pathogen-associated molecular patterns reduction, modification, and hiding to ensure low stimulatory activity and toxicity for cells [3]. This strategy allows *Brucella* to reach its replication niche before activating antimicrobial mechanisms by host immune responses. However, inside the host cells, *Brucella* releases vital molecules for the bacteria that trigger the activation of host cytosolic receptors [4,5]. In the paper by Tupik et al. [6], in vivo studies using *Asc*^−/−^ mice infected with *Brucella* revealed an increased bacteria load and decreased immune cell recruitment. The findings of the study suggest that the protective role of ASC may result from the induction of pyroptosis through a gasdermin D-dependent mechanism in macrophages. However, further studies are required to elucidate this complex circuit by which the host immune system recognizes *Brucella-*derived molecules. This editorial summarizes the data described in the Special Issue entitled “Host Immune Response and Pathogenesis to *Brucella* spp. Infection” consisting of seven research articles and two reviews. These contributions report several aspects of host–*Brucella* interactions, and these findings will help to advance the comprehension of bacterial pathogenesis and contribute to the future development of drugs or vaccines to control brucellosis.

For a successful infection process, a pathogen has to invade, survive, and replicate in host cells. During the first steps of *Brucella* trafficking, the bacteria is able to block the progression of its cell cycle, remaining at the G1 stage for several hours, before it reaches its replication niche. The work of Van der Henst et al. [7] demonstrated that starvation mediated by guanosine tetra- or penta-phosphate, (p)ppGpp, is one of the factors contributing to G1 arrest observed in *B. abortus* infection in macrophages. Adhesion to target cells is another major step forward for bacterial invasion and replication. Bialer et al. [8] reviewed the *Brucella* adhesins and their role in mediating adhesion to cells. These molecules include the sialic acid-binding proteins SP29 and SP41 (binding to erythrocytes and epithelial cells, respectively), the BigA and BigB proteins that contain an Ig-like domain (binding to cell adhesion molecules in epithelial cells), the monomeric autotransporters BmaA, BmaB, and BmaC (binding to extracellular matrix components, epithelial cells, osteoblasts, synoviocytes, and trophoblasts), the trimeric autotransporters BtaE and BtaF (binding to ECM components and epithelial cells), and Bp26 (binding to ECM components). After binding, replication in phagocytic and non-phagocytic cells is required to establish infection. One of the main clinical signs of brucellosis is abortion in domestic animals. Zavattieri et al. [9] demonstrate that *Brucella abortus* was able to infect and survive in both non-decidualized and decidualized human endometrial stromal cells (T-HESC cell line). *Brucella* infection did not induce cytotoxicity and did not alter the decidualization status of cells, but elicited the secretion of IL-8 and MCP-1 in either decidualized or non-decidualized T-HESC. The proinflammatory responses induced by *Brucella* infection in T-HESC may contribute to the gestational complications and abortion during brucellosis.

Brucellae reside mostly within phagocytes and other cells, including trophoblasts, where they establish a preferred replicative niche inside the endoplasmic reticulum. González-Espinoza et al. [10] propose that *Brucella* takes advantage of the environment provided by the cellular niches in which it resides to generate reservoirs and disseminate to other organs, such as spleen, lymph nodes, liver, bone marrow, epididymis, and placenta. They discuss in this review how the favored cellular niches for *Brucella* infection in the host give rise to anatomical reservoirs that may lead to chronic infections or persistence in asymptomatic subjects, and which may be considered a threat for further contamination. The natural infection by *Brucella* occurs mainly by oral and nasal routes through the consumption of raw milk and unpasteurized dairy products from infected animals, the inhalation of aerosols containing the pathogen, and/or contact with infected animals and their secretions. Considering that the oral route is the main route of natural infection in humans and animals, there is a need to understand the mechanisms of the establishment of oral infection so that new therapeutic strategies can be developed in order to control this disease. Santos et al. [11] report the role of ST2 receptor in a murine model of oral infection with *Brucella abortus* and its influence on gut homeostasis and control of bacterial replication. Their results suggest that ST2^−/−^ are more resistant to *B. abortus* infection, as lower bacterial CFUs were detected in the livers and spleens of knockout mice when compared to wild-type Additionally, they observed an increase in intestinal permeability in WT infected mice compared to ST2^−/−^ animals. Finally, their findings suggest that ST2 receptor is involved in the invasion process of *B. abortus* by the mucosa in the oral infection model.

Regarding pathology, the most frequent clinical characteristics of brucellosis besides abortion are hepatomegaly, splenomegaly, and peripheral lymphadenopathy, revealing the preference of *Brucella* for the reticuloendothelial system. The research article by Arriola-Benitez et al. [12] describes how *Brucella abortus* infection induces the upregulation of class II transactivator protein (CIITA) with concomitant MHC-I and -II expression in immortalized human hepatic stellate cell line (LX-2) in a manner that is independent from the expression of the type 4 secretion system (T4SS). Since hepatocytes constitute the most abundant epithelial cell in the liver, experiments were conducted to determine the contribution of these cells in antigen presentation in the context of *B. abortus* infection. The results indicated that *B. abortus*-infected hepatocytes have an increased MHC-I expression, but MHC-II levels remain at basal levels. Overall, the authors revealed that *B. abortus* infection of hepatic stellate cells and hepatocytes is able to differentially regulate the MHC expression, thus stimulating the T-cell specific-immune response in the liver. The central nervous system (CNS) invasion by bacteria of the genus *Brucella* results in an inflammatory disorder termed neurobrucellosis. The precise mechanism whereby the bacterium leaves the bloodstream and gains access to the CNS remains unclear. Regardless of the mechanism, it is clear that once the bacterium reaches the CNS it induces a pathological pro-inflammatory response. The study of Rodriguez et al. [13] investigated the role of *Brucella abortus*-stimulated platelets on human brain microvascular endothelial cell (HBMEC) activation. Platelets enhanced HBMEC activation in response to *B. abortus* infection. Additionally, supernatants from *B. abortus*-activated platelets promoted the transendothelial migration of neutrophils and monocytes depending on the Erk1/2 signaling pathway. The results of this study describe a mechanism whereby *B. abortus*-stimulated platelets induce endothelial cell activation, promoting neutrophils and monocytes to traverse the blood–brain barrier, probably contributing to the inflammatory pathology of neurobrucellosis.

Vaccination is the major countermeasure to control *Brucella* infection. Currently used *Brucella* vaccines, *Brucella abortus* strain 19 and RB51, are comprised of live attenuated *Brucella* strains and prevent infection in animals. The study of Gupta et al. [14] tested the recombinant proteins Omp25 and L7/L12 as potential vaccine candidates. Challenge with virulent *B. abortus* 544 demonstrated that Omp25+L7/L12-vaccinated mice exhibited superior log10 protection (1.98) compared to individual vaccines L7/L12 (1.75) and Omp25 (1.46). However, further studies are necessary to test the effectiveness of this divalent vaccine in large animals. Additionally, the cost of recombinant vaccines has to be taken into account when discussing veterinary vaccines. The articles included in this Special Issue present novel data on *Brucella* spp. infections, including host immune responses and bacterial pathogenesis that contribute significantly to improving the understanding of this disease.
